# Assessment of intrinsic and extrinsic signaling pathway in excitotoxic retinal ganglion cell death

**DOI:** 10.1038/s41598-018-22848-y

**Published:** 2018-03-15

**Authors:** Berkeley K. Fahrenthold, Kimberly A. Fernandes, Richard T. Libby

**Affiliations:** 1 Department of Ophthalmology, University of Rochester Medical Center, Rochester, NY 14642 USA; 2 Neuroscience Graduate Program, University of Rochester Medical Center, Rochester, NY 14642 USA; 3 Department of Biomedical Genetics, University of Rochester Medical Center, Rochester, NY 14642 USA; 4 The Center for Visual Sciences, University of Rochester Medical Center, Rochester, NY 14642 USA

## Abstract

Excitotoxicity leads to the activation of a cytotoxic cascade that causes neuronal death. In the retina, retinal ganglion cells (RGCs) die after an excitotoxic insult. Multiple pathways have been proposed to contribute to RGC death after an excitotoxic insult, including TNF signaling, JNK activation, and ER stress. To test the importance of these pathways in RGC death after excitotoxic injury, the excitotoxin N-methyl-D-aspartate (NMDA) was intravitreally injected into mice deficient in components of these pathways. Absence of *Tnf* or its canonical downstream mediator, *Bid*, did not confer short- or long-term protection to RGCs. Despite known activation in RGCs and a prominent role in mediating RGC death after other insults, attenuating JNK signaling did not prevent RGC death after excitotoxic insult. Additionally, deficiency of the ER stress protein DDIT3 (CHOP), which has been shown to be involved in RGC death, did not lessen NMDA induced RGC death. Furthermore, absence of both *Jun* (JNK’s canonical target) and *Ddit3*, which together provide robust, long-term protection to RGC somas after axonal insult, did not lessen RGC death. Collectively, these results indicate that the drivers of excitotoxic injury remain to be identified and/or multiple cell death pathways are activated in response to injury.

## Introduction

Excessive stimulation of glutamate receptors has been shown to disrupt the intracellular environment and lead to activation of cytotoxic cascades culminating in neuronal death^1^. In the retina, excitotoxic insult is thought to contribute to several diseases, including diabetic retinopathy and retinal ischemia^2–4^. Multiple cell types, including both neurons and glia, are affected by excitotoxic insult. In the retina, excitotoxic insult is known to cause the death of retinal ganglion cells (RGCs) and at least some types of amacrine cells^5–9^. Despite excitotoxicity’s importance in disease, the required cell death pathways controlling excitotoxic induced RGC death are not well-defined.

Multiple intrinsic and extrinsic cell death signaling pathways have been implicated in neuronal death after excitotoxic injury. Recently, TNFR1 activation has been shown to initiate the process of RGC death after excitotoxic insult^7^. A key mediator downstream of TNFR1 activation, the pro-apoptotic protein BID, has also been shown to be important in mediating neuronal damage after glutamate toxicity^10^ including in RGCs^11^. Interestingly, if BID is important for NMDA induced RGC death, it is not acting through BAX activation, since *Bax* deficiency does not prevent RGC death after excitotoxic insult^8,12^. Additionally, the JNK (c-Jun N-terminal kinases) pathway has been proposed as a regulator of neuronal death after excitotoxic insult. Canonical JNK signaling leads to activation of the transcription factor JUN, a member of the activation protein-1 (AP-1) family of transcription factors^13^. In the eye, JNK and JUN activation in RGCs is present after induced ischemia, axonal injury, increased intraocular pressure, and excitotoxicity^14–19^. Pan inhibitors against JNK, as well as *Jun* antisense oligodeoxynucleotides, provided some protection to inner retinal neurons after an excitotoxic insult^20–22^. Furthermore, ER stress has been proposed to mediate excitotoxic cell death^23–26^. In the retina, an increase in expression of the ER stress gene *Ddit3* (*Chop*) has been found in RGCs after intravitreal injection of the excitotoxin NMDA^25^. *Ddit3* deficiency provided a modest, but significant level of protection of ganglion cell layer neurons (which consists of RGCs and amacrine cells) after and intravitreal injection of NMDA^23^. It has also been observed that lack of *Ddit3* significantly reduces RGC death after axonal injury^27^. Thus, there appears to be numerous cell death pathways contributing to RGC death after an excitotoxic insult. Here, the importance of these pathways in excitotoxic RGC death is critically tested using mice deficient in critical molecular components of these pathways.

## Results

### Intravitreal NMDA injection kills RGCs

**Figure 1:**
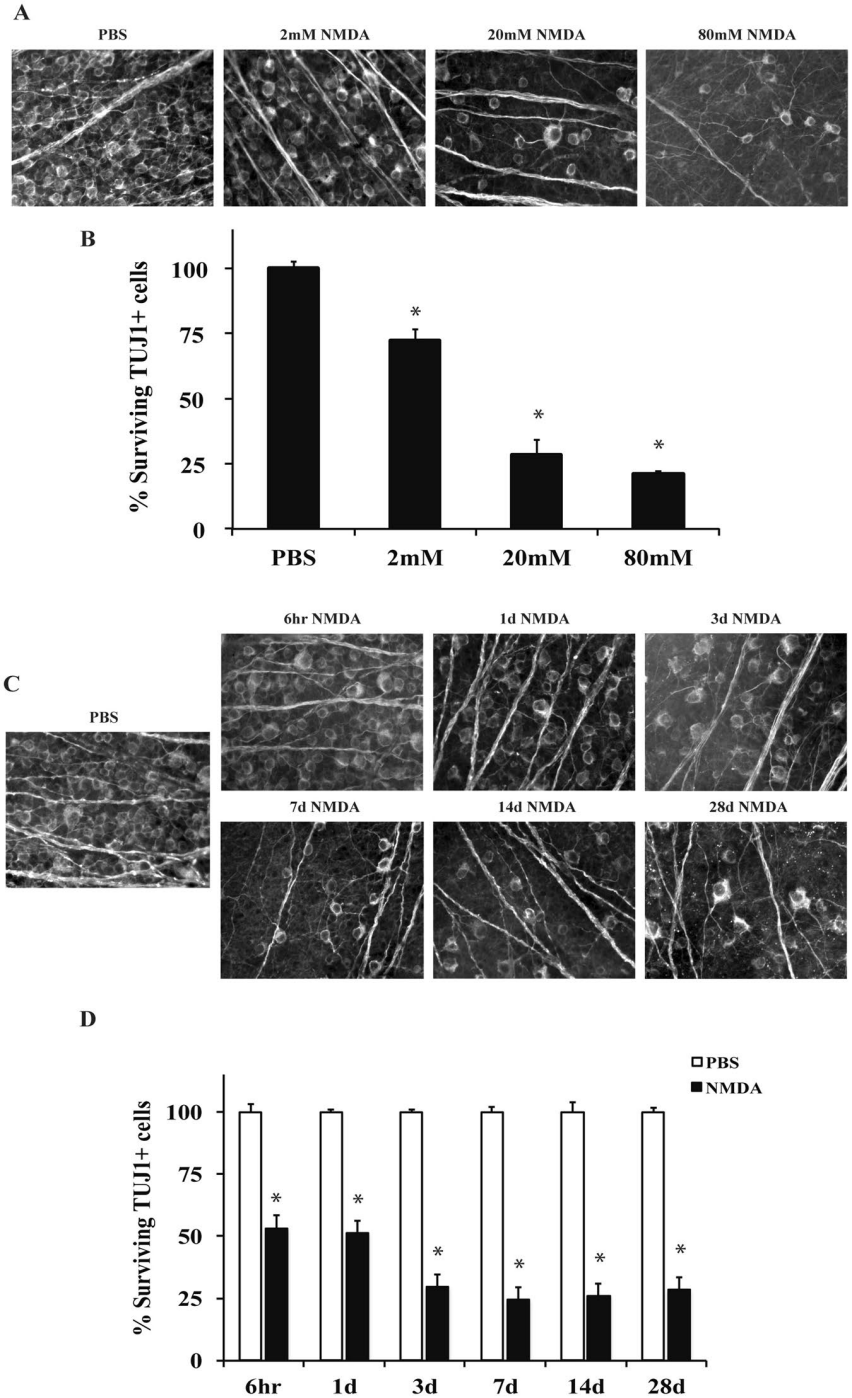
NMDA induced excitotoxic insult kills RGCs in a dose dependent manner. RGC loss was quantified 7 days after intravitreal injection of NMDA. (**A**,**B**) The RGC specific marker, TUJ1, was used to label RGCs in flat-mounted retinas 7 days after a 2 μl intravitreal injection of either PBS (vehicle control) or 2 mM, 20 mM, or 80 mM of NMDA into C57BL/6 J mice (n = 23 for PBS control, n = 6 for 2 mM NMDA, n = 6 for 20 mM NMDA, n = 3 for 80 mM NMDA). Quantification of TUJ1+ RGCs showed NMDA insult caused a dose dependent loss of RGCs. All NMDA concentrations caused a significant decrease in RGC number (*p < 0.001 for all comparisons). Note, that there was no significant difference in RGC number between the 20 mM and 80 mM concentrations (*p* = 0.819). (**C**,**D**) 20 mM NMDA was shown to induce greater than 50% RGC death at 7 days and there appeared to be no benefit to increasing the dose. Thus, 20 mM of NMDA was chosen to investigate the molecular pathways controlling NMDA induced RGC death. To understand the time course of RGC death induced by 20 mM of NMDA, TUJ1+ cells were counted in flat-mounted retinas 6hr, 1d, 3d, 7d, 14d, and 28d after (n ≥ 5 for all time points). NMDA injection resulted in significant loss of RGCs compared to controls at all time points examined (*p < 0.001). Note, approximately 50% of TUJ1 + cells were lost after 6 hours and after 3 days, no more apparent loss of TUJ1+ cells. Scale bar: 50 μm.

Administration of excitotoxins to the retina affects multiple cell types, including RGCs^28^. In order to establish a relevant concentration of NMDA to study NMDA-induced RGC death, the effect on RGC survival of different concentrations of NMDA was examined. 2 μl of three concentrations of NMDA (2 mM, 20 mM, or 80 mM) were intravitreally injected into C57BL/6 J mice. For controls, the contralateral eye was injected with 2 μl of PBS. The number of TUJ1+ RGCs remaining 7 days after injection was counted. All concentrations of NMDA (2 mM, 20 mM and 80 mM) resulted in a significant decrease in the number of TUJ1+ RGCs compared to control eyes (*p* < 0.001 for all concentrations compared to control; Fig. 1A,B). There was also significant decrease in cell number between 2 mM and 20 mM injections of NMDA (*p* < 0.001, for all comparisons); however, there was no significant decrease in cell number between 20 mM and 80 mM NMDA injection (*p* = 0.819). The 20 mM NMDA concentration was chosen for future experiments based upon this concentration producing a significant amount of retinal neuron loss and relevance to previously published studies^7,29–31^. To determine the window of RGC loss, TUJ1+ cells were counted 6hrs, 1d, 3d, 7d, 14d, and 28d after intraocular injection of 20 mM NMDA (Fig. 1C,D). There was a significant loss of approximately 50% of TUJ1+ cells 6 hr and 1d after NMDA insult (*p* < 0.001 comparing either 6 hour or 1 day after NMDA injection to control eyes). Between 1 day and 3 days after injection there was a further loss of RGCs (*p* = 0.007, comparing 1d to 3d). However, after 3 days, there was not a further, significant decrease in TUJ1+ cell number *(p* > 0.05 comparing 3d to either 7d, 14d, or 28d). Thus, cell loss appears to be complete by 3 days after 20 mM NMDA insult.

### TNF and BID are not required for NMDA-induced RGC death

**Figure 2:**
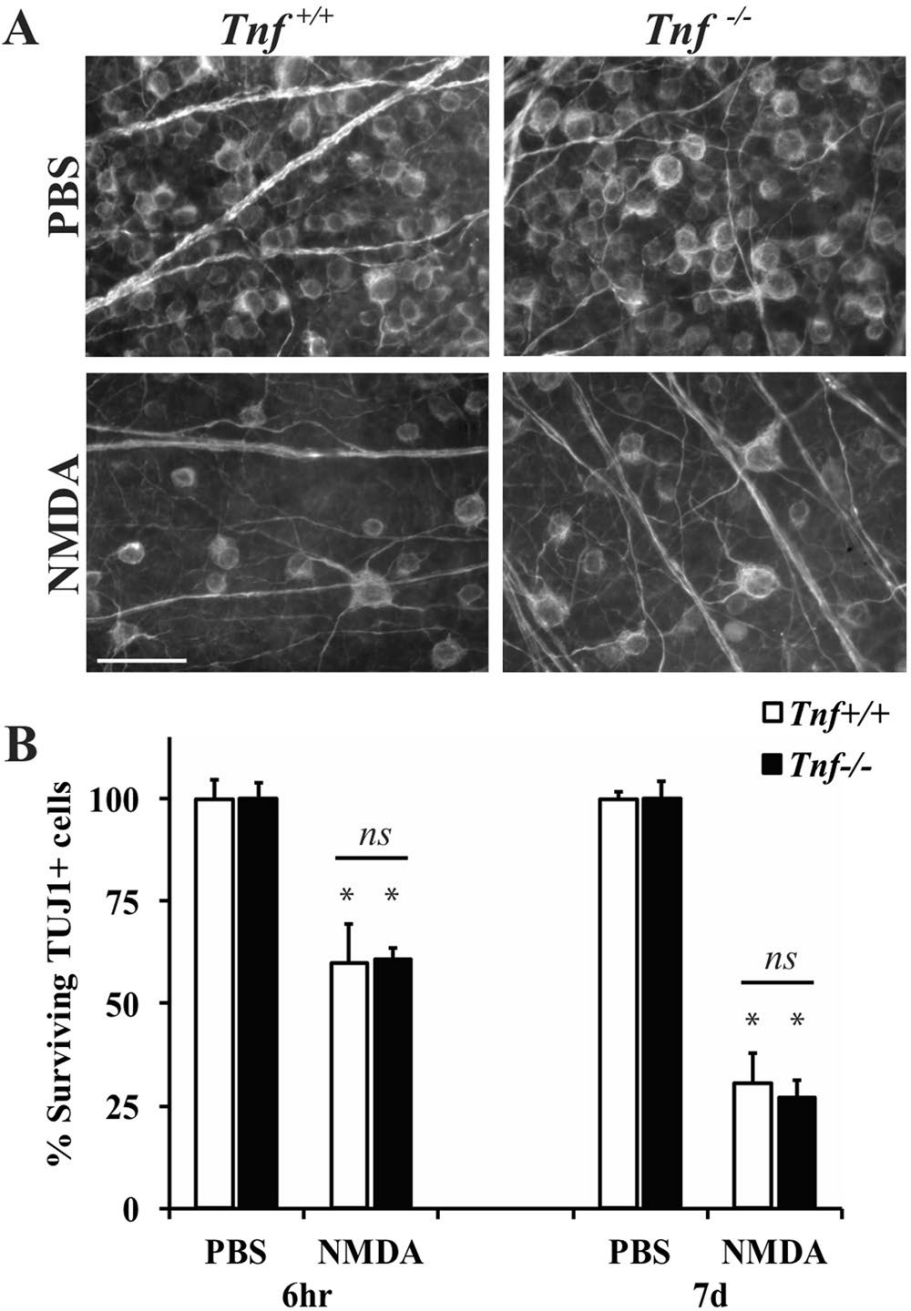
*Tnf* deficiency does not prevent RGC death after excitotoxic insult. To determine if TNF is required for NMDA induced RGC death, 2 μl PBS or 20 mM NMDA was injected into the vitreous of wildtype (*Tnf*^+/+^) and *Tnf* deficient (*Tnf*^−/−^) mice. The number of surviving RGCs (TUJ1 + cells) were counted 6 hours (hr) and 7 days (d) after intravitreal NMDA injection (n = 5 for 6 hr PBS *Tnf*^+/+^, n = 5 for 6 hr NMDA *Tnf*^+/+^, n = 5 for 6hr PBS *Tnf*^−/−^, n = 5 for 6hr NMDA *Tnf*^−/−^, n = 6 for 7d PBS *Tnf*^+/+^, n = 7 for 7d NMDA *Tnf*^+/+^, n = 5 for 7d PBS *Tnf*^−/−^, n = 5 for 7d NMDA *Tnf*^−/−^). (**A**) Representative images from flat mounted retinas stained with anti-TUJ1 show clear RGC loss in both wildtype and *Tnf* deficient mice after NMDA insult at both time points examined. (**B**) Quantification of TUJ1+ RGCs showed a significant loss of TUJ1 + cells in both wildtype and *Tnf* deficient mice at both 6 hours and 7 days after injury (*p < 0.001 for PBS to NMDA comparisons, P = 0.9694 comparing WT to *Tnf*^−/−^ NMDA). Note, there was no difference in RGC number between *Tnf* wildtype and deficient mice at either time *(p* > 0.9 for each time point). Scale bar: 50 μm.

TNF has been suggested to be critical for inducing RGC death 6 hours after excitotoxic injury^7^. In order to determine if TNF is required for long-term NMDA induced RGC death, 2 µl of PBS (control, contralateral eye) or 20 mM of NMDA was injected into the vitreous of *Tnf* deficient (*Tnf*^−/−^) mice. The number of surviving TUJ1+ RGCs was counted at 6 hours and 7 days after intravitreal NMDA injection, two time points that encompass when RGCs are dying and after the time window of cell death (see Fig. 1). *Tnf* deficiency did not lessen RGC loss at either the 6 hour or 7 day time point compared to wildtype mice (*p* = 0.9694 comparing WT to *Tnf*^−/−^ NMDA; Fig. 2). These data suggest that *Tnf* is not required nor an important contributor to RGC death after excitotoxic injury.

**Figure 3:**
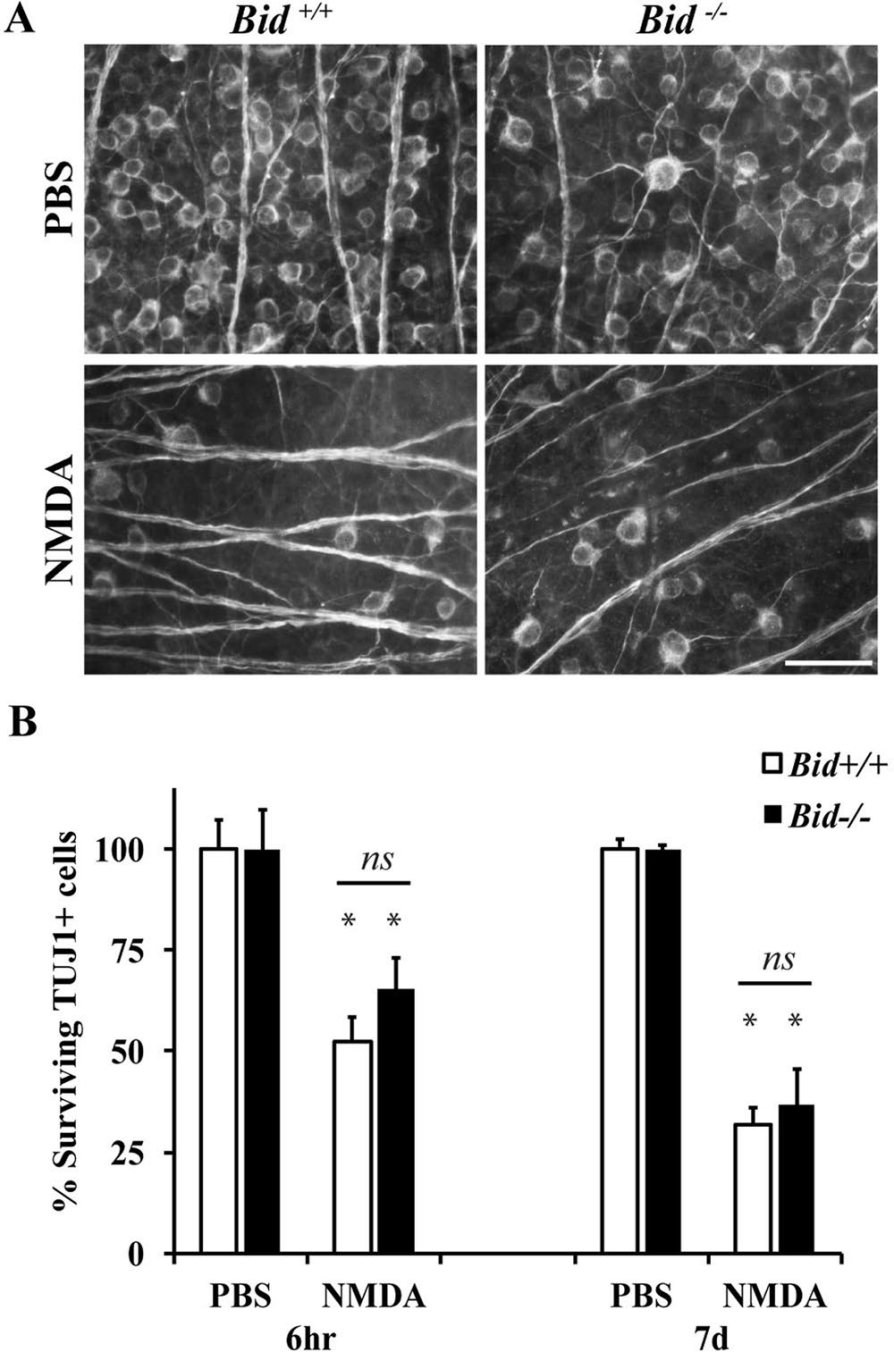
*Bid* deficiency does not prevent RGC death after excitotoxic insult. BID is a major regulator of extrinsically-induced cell death. To determine if BID is required for NMDA induced RGC death, 2 μl PBS or 20 mM NMDA was intravitreally injected into wildtype *Bid* (*Bid*^+/+^) and *Bid* deficient (*Bid*^−/−^) mice. The number of surviving RGCs (TUJ1 + cells) were counted 6 hours (hr) and 7 days (d) after intravitreal NMDA injection (n = 5 for 6 hr PBS *Bid*^+/+^, n = 5 for 6 hr NMDA *Bid*^+/+^, n = 5 for 6 hr PBS *Bid*^−/−^, n = 5 for 6 hr NMDA *Bid*^−/−^, n = 6 for 7d PBS *Bid*^+/+^, n = 6 for 7d NMDA *Bid*^+/+^, n = 7 for 7d PBS *Bid*^−/−^, n = 7 for 7d NMDA *Bid*^−/−^). (**A**) Representative images from flat mounted retinas stained with anti-TUJ1 show clear RGC loss in both wildtype and *Bid* deficient mice after NMDA insult at both time points examined. (**B**) Quantification of TUJ1 + RGCs showed a significant loss of TUJ1+ cells in both wildtype and *Bid* deficient mice at both 6 hours and 7 days after injury (*p < 0.001 for PBS to NMDA comparisons). Note, there was no difference in RGC number between *Bid* wildtype and deficient mice at either time (*p* ≥ 0.719 for each time point). Scale bar: 50 μm.

Extrinsic death receptors, including TNFR1, may recruit the intrinsic pathway in order to amplify the cell death cascade. This is mediated through BID cleavage and fragment translocation to the mitochondria where it activates BAX and/or BAK, two pro-apoptotic BCL2 proteins that are critical for apoptotic cell death^32^. To determine if BID is required for NMDA induced RGC death, 2 μl of either 20 mM NMDA or PBS was injected into the vitreous of *Bid* deficient (*Bid*^−/−^) and wildtype (*Bid*^+/+^) mice. The number of surviving RGCs (TUJ1+ cells) was counted at 6 hours and 7 days after insult. Significant RGC loss in both wildtype and *Bid* deficient eyes was observed at both time points. Furthermore, *Bid* deficiency did not lessen RGC death (Fig. 3; *p* ≥ 0.719 comparing WT to *Bid*^−/−^ NMDA). These data suggest BID is not a critical mediator of short- and long-term excitotoxic RGC death.

### JUN is activated in multiple different retinal cell types after excitotoxic insult

**Figure 4:**
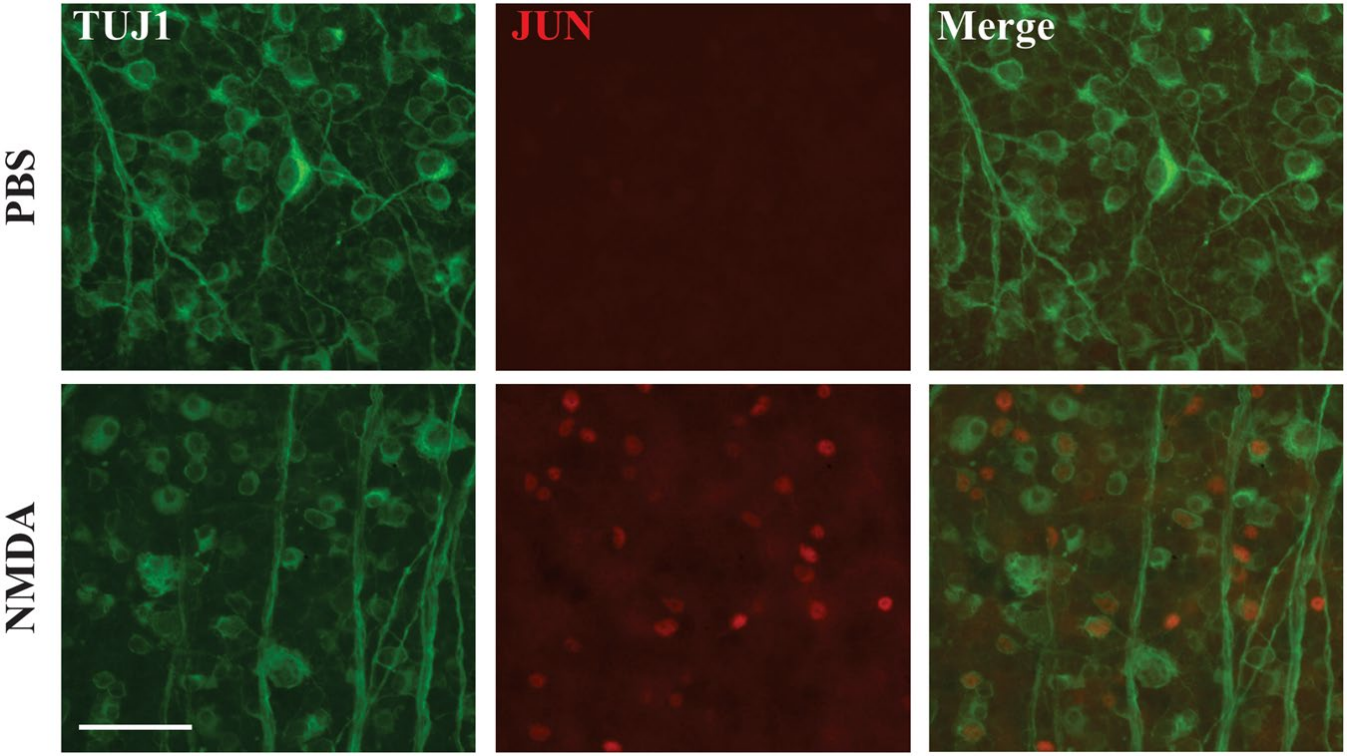
JUN signaling is activated in retinal cells *in vivo* following intravitreal injection of NMDA. Excitotoxic injury causes JUN activation as represented by pJUN in retinal cells, specifically RGCs, 6 hours after intravitreal injections of either NMDA. Representative images of retinal flat mounts stained with TUJ1 and JUN 6hrs after NMDA injection, showing activation of JUN in TUJ1 + cells (n = 3 for each condition). Scale bar, 50 μm.

The transcription factor JUN is activated by JNK signaling. JNK inhibitors have lessened RGC death after an NMDA insult^20–22^. However, there was variability in RGC protection between inhibitors and it is probable that the inhibitors are not completely specific to JNKs, therefore it is important to test the JNK pathway in excitotoxicity induced RGC death using genetics. In order to determine if JUN was activated (phosphorylated) in retinal cells after an excitotoxic insult, retinal flat mounts were examined for pJUN at 6 hours after intravitreal injections of either NMDA or PBS. Similar to previous findings, pJUN was detected in TUJ1+ RGCs after NMDA insult (Fig. 4)^22^. These results demonstrate that JUN dependent MAPK signaling is active in RGCs after an NMDA insult.

### MAPK signaling does not appear to be important for RGC death after NMDA insult

**Figure 5:**
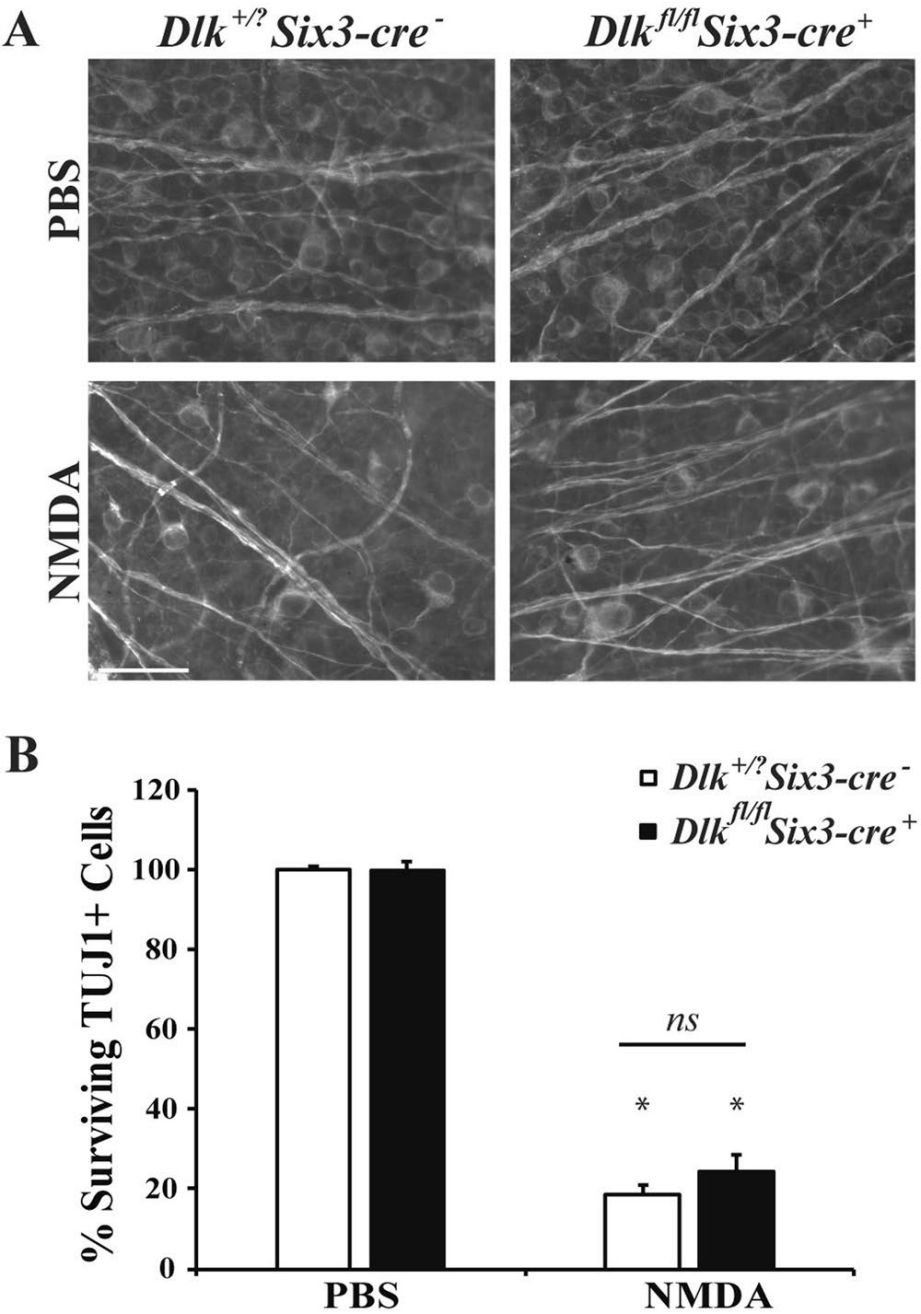
*Dlk* deficiency does not protect RGCs from NMDA-induced death. (**A**) Representative images showing TUJ1 immunolabeled cells in the GCL of control (*Dlk*^+/+^*; Six3-cre*^*−*^ or *Dlk*^+*/fl*^*; Six3-cre*^*−*^) *Dlk* deficient (*Dlk*^*fl/fl*^*; Six3-cre*^+^) mice 7 days after intravitreal NMDA or PBS injection (n = 4 for PBS WT; n = 3 for NMDA WT, n = 5 for PBS *Dlk*^*fl/fl*^*; Six3-cre*^+^*, n* = 4 for NMDA *Dlk*^*fl/fl*^*; Six3-cre*^+^). There was significant RGC loss in both control and *Dlk* deficient mice after NMDA insult. (**B**) Quantification of TUJ1 + RGCs showed that *Dlk* deficient mice had a similar loss of RGCs compared to wildtype mice 7d after NMDA injection (*p < 0.001 for comparison of PBS to NMDA; *p* ≥ 0.1843 for comparison between genotypes, *ns* between genotypes). Scale bar: 50 μm.

DLK is a MAP3K upstream of JNK and JUN that has been shown to be critical for excitotoxic-induced neuronal death. Deficiency of *Dlk* significantly protects CA1 hippocampal neurons after excitotoxic injury^33^. In order to assess the involvement of DLK in excitotoxic mediated RGC loss, DLK was conditionally deleted in the retina using a floxed allele of *Dlk* and the retinal cre, *Six3-cre*. Previously, we have shown that *Six3*-*cre* successfully deletes *Dlk* (and *Jun*, discussed below) in approximately 85% of RGCs^34,35^. Seven days post-NMDA injection, RGC loss was comparable between wildtype and *Dlk* deficient (*Dlk*^*fl/fl*^*; Six3-cre*^+^) mice (Fig. 5; *p* ≥ 0.1843 comparing WT to *Dlk*^*fl/fl*^*; Six3-cre*^+^ NMDA injected eyes). These results suggest that DLK is not critical for NMDA-induced RGC death.

**Figure 6:**
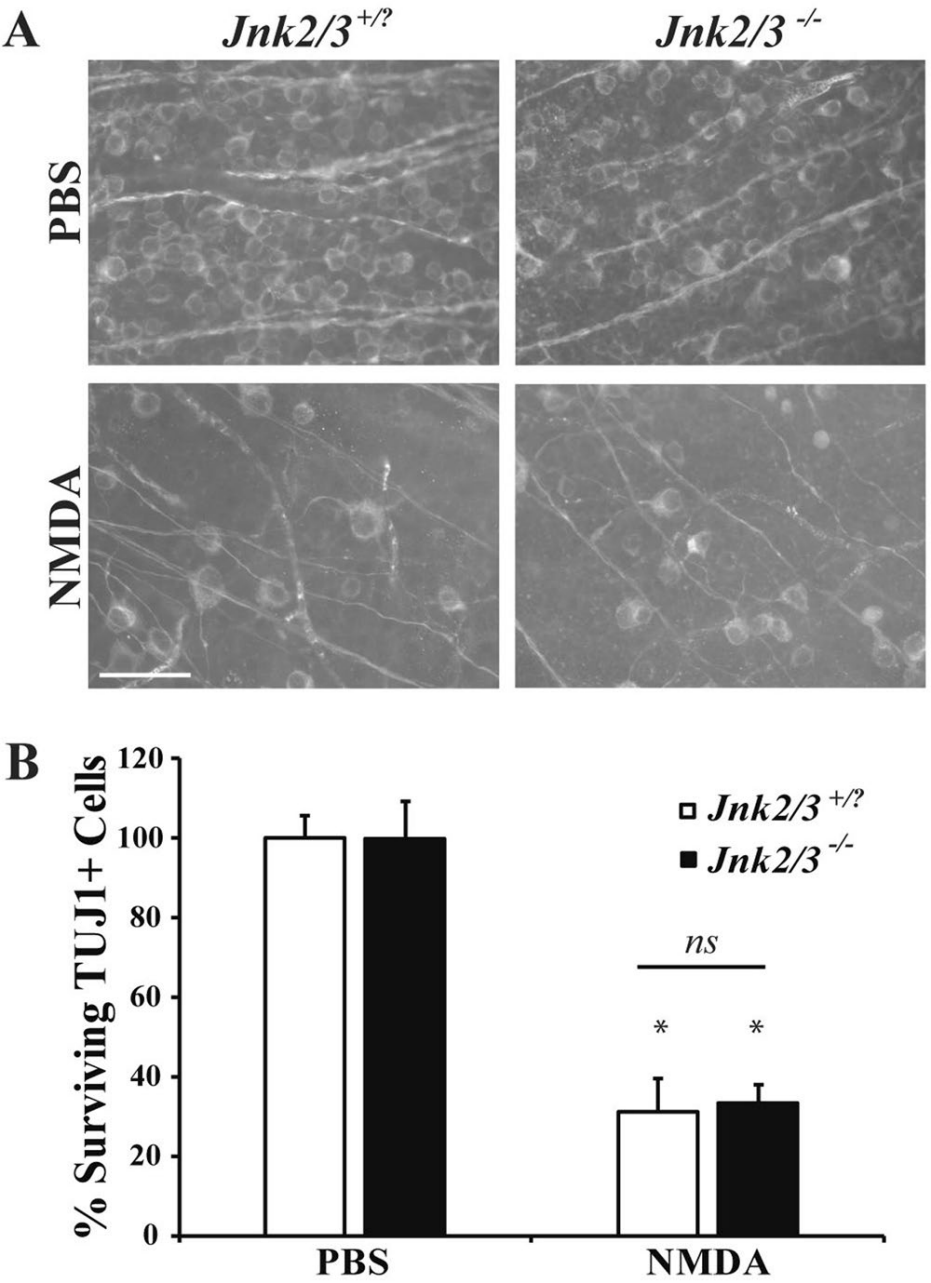
*Jnk2*^−/−^*Jnk3*^−/−^ nulls do not have attenuated RGC loss after excitotoxic injury. (**A**) Representative images showing TUJ1 immunolabeled cells in the GCL of *Jnk2Jnk3* deficient mice 7 days after intravitreal NMDA or PBS injection, revealing significant RGC loss in both wildtype (*Jnk2*^+*/?*^*Jnk3*^+*/?*^) and *Jnk2*^−/−^*Jnk3*^−/−^ deficient mice after NMDA insult (n = 4 for PBS WT, n = 4 for NMDA WT, n = 3 for PBS *Jnk2*^−/−^*Jnk3*^−/−^*, n* = 3 for NMDA *Jnk2*^−/−^*Jnk3*^−/−^). (**B**) Quantification of TUJ1 + RGCs confirmed that *Jnk2*^−/−^*Jnk3*^−/−^ deficient mice had a similar loss of RGCs compared to wildtype mice 7d after NMDA injection (*p ≤ 0.001 for comparison between PBS and NMDA; *p* ≥ 0.9769 comparing WT to *Jnk2*^−/−^*Jnk3*^−/−^ NMDA; *ns* between genotypes; two-way ANOVA, Tukey’s multiple comparisons test). Scale bar: 50 μm.

There are three JNKs in vertebrates, *Jnk1, Jnk2*, and *Jnk3*^36^. Deficiency of *Jnk3* has been reported to confer protection to hippocampal neurons in the brain after excitotoxic injury^37^. Inhibitors against JNK have produced mixed results, showing minimal or very significant protection for RGCs after excitotoxicity^20,22^. After axonal injury, combined absence of *Jnk2* and *Jnk3* provides significant protection from RGC loss, showing that *Jnk2* and *Jnk3* can mediate pro-death signaling in injured RGCs^38^. Therefore, in order to examine the role of JNK in NMDA mediated RGC death using a genetic approach, mice deficient for both *Jnk2* and *Jnk3* (*Jnk2*^−/−^ *Jnk3*^−/−^) were intravitreally injected with 2 μl of 20 mM NMDA. Compared to wildtype mice, the number of RGCs lost 7 days post-intravitreal injection of NMDA was similar in *Jnk2*^−/−^ *Jnk3*^−/−^ mice (Fig. 6; *p* ≤ 0.001 for comparison between PBS and NMDA for each genotype, *p* ≥ 0.9769 comparing WT to *Jnk2*^−/−^ *Jnk3*^−/−^ NMDA). This result shows that the JNK involved in excitotoxic death in other neurons (JNK3) and the JNKs that have been shown to be important in RGC death after an insult (JNK2 and JNK3) are not required for NMDA-mediated RGC death. This suggests that *Jnk1* could have a role in the excitotoxic injury response, in the future it would be interesting to look at a conditional knockout of all three JNK isoforms.

**Figure 7:**
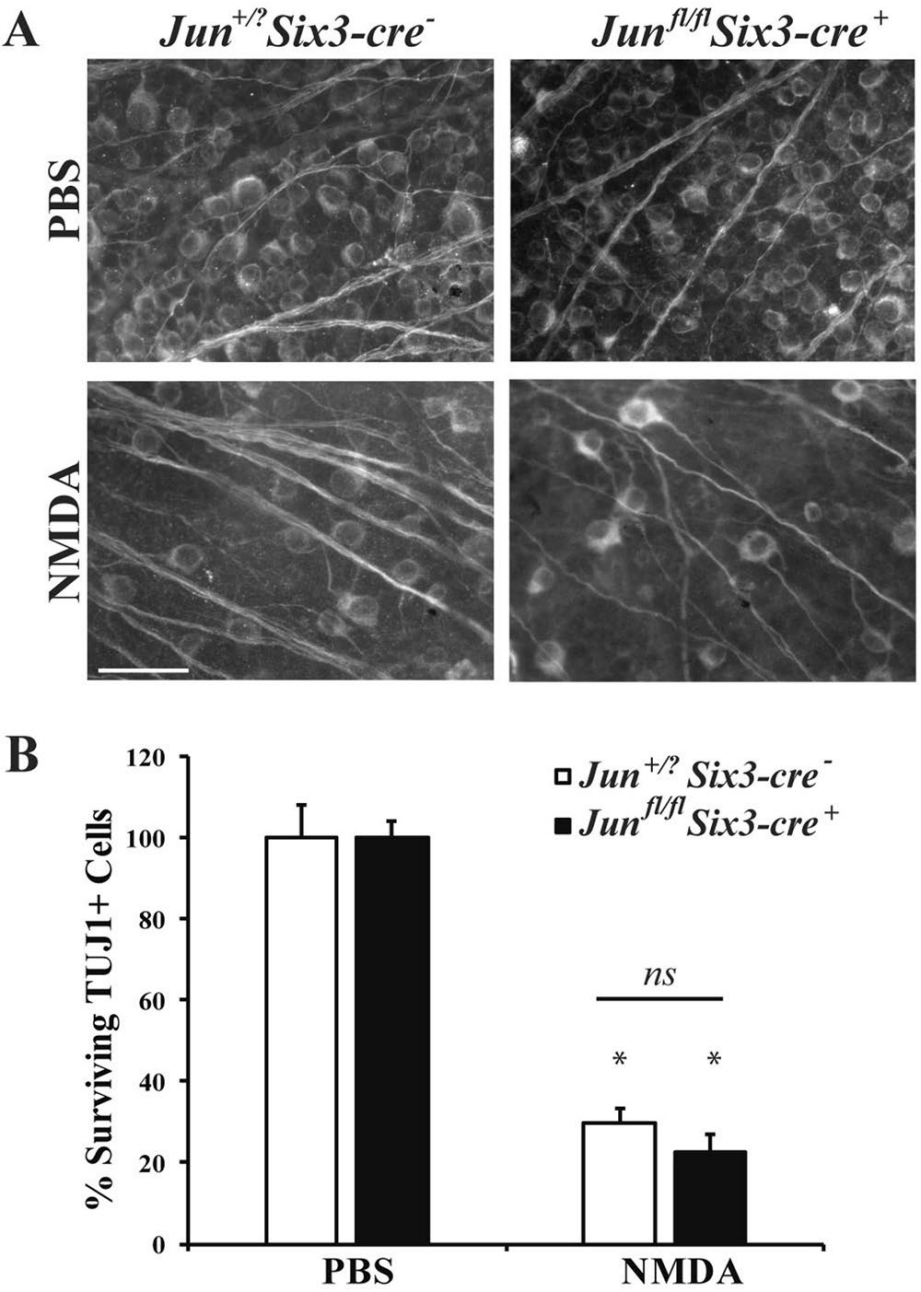
Deficiency of JUN does not protect RGCs after excitotoxic injury. To determine if JUN is required for NMDA induced RGC death, NMDA was injected into the vitreous of *Jun* deficient (*Jun*^*fl/fl*^*; Six3-cre*^+^) mice. The number of surviving RGCs (TUJ1+ cells) were counted 7 days after intravitreal NMDA injection. (**A**) Representative images from flat mounted retinas stained with anti-TUJ1 show significant RGC loss in both wildtype (*Jun*^+*/?*^*; Six3-cre*^−^) and *Jun*^*fl/fl*^*; Six3-cre*^+^ mice after NMDA insult (n = 5 for PBS WT, n = 5 for NMDA WT, n = 6 for PBS *Jun*^*fl/fl*^*; Six3-cre*^+^, n = 5 for NMDA *Jun*^*fl/fl*^*; Six3-cre*^+^). (**B**) Quantification of TUJ1+ RGCs confirmed that *Jun* deficient mice had a similar loss of RGCs compared to wildtype mice 7d after NMDA injection (*p < 0.001, comparing 20 mM NMDA to PBS control; *p* = 0.9846 comparing WT to *Jun*^*fl/fl*^*; Six3-cre* NMDA; *ns* between genotypes). Scale bar: 50 μm.

JUN is a canonical injury response factor downstream of JNK and DLK that is known to mediate RGC death. JUN has been shown to be a mediator of RGC death after other injuries, for example inhibiting *Jun* significantly lessens RGC death after mechanical axonal injury or axotomy^38,39^. The role of JUN in excitotoxic-mediated RGC death is unclear, though protection of RGCs was found using a c-Jun antisense oligodeoxynucleotide after excitotoxic insult^21^. In order to critically test the importance of JUN in NMDA induced RGC death, NMDA was injected into the vitreous of *Jun* deficient mice. Deletion of *Jun* is embryonically lethal, therefore a conditional retinal deletion of JUN was achieved using a floxed allele of JUN and the early retinal deleter cre, *Six3-cre* (*Jun*^*fl/fl*^*; Six3-cre*^+^; referred to as *Jun* deficient mice)^40–43^. The number of surviving RGCs (TUJ1+ cells) were counted 7 days after intravitreal injection, a time point when the majority of RGCs have died in wildtype retinas. There was a significant loss of RGCs in both the wildtype and *Jun* deficient retinas after NMDA insult (Fig. 7; *p* = 0.9846, comparing WT to *Jun*^*fl/fl*^*; Six3-cre*^+^ NMDA). This result shows that the injury response factor JUN is not required for NMDA-mediated RGC death.

### The ER stress protein Ddit3 (Chop) is not required for RGC excitotoxic death

**Figure 8:**
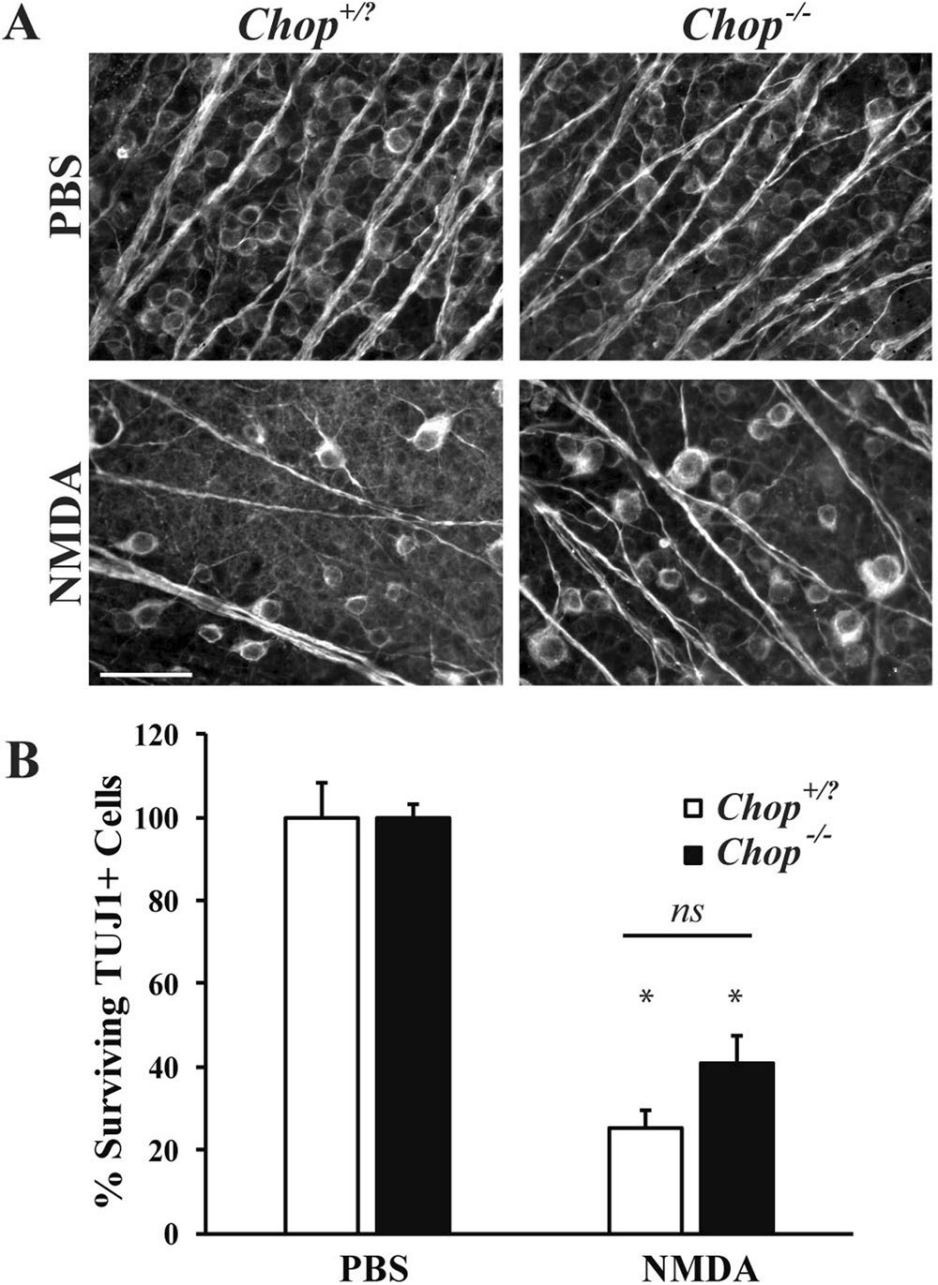
*Ddit3* deficiency provides modest protection of RGCs after NMDA injury. (**A**) Representative images showing TUJ1 immunolabeled cells in the GCL of *Ddit3* deficient (*Ddit3*^−/−^), mice 7 days after intravitreal NMDA or PBS injection. Significant RGC loss occurred in wildtype (*Ddit3*^+*/?*^), but *Ddit3* deficient mice exhibited modest protection of RGCs after NMDA insult (n = 3 for PBS WT, n = 3 for NMDA WT, n = 8 for PBS *Ddit3*^−/−^, n = 10 for NMDA *Ddit3*^−/−^). (**B**) Quantification of TUJ1+ RGCs confirmed that *Ddit3* deficient mice had significantly more surviving RGCs compared to wildtype mice 7d after NMDA injection (*p < 0.001 comparing PBS to NMDA; *p* = 0.8105 comparing WT to *Ddit3*^−/−^ NMDA; *ns* between genotypes). Scale bar: 50 μm.

ER stress has been suggested to mediate excitotoxic RGC death and an increase in expression of the ER stress protein *Ddit3* has been found in RGCs after NMDA injury^25^. *Ddit3* has been shown to be involved in RGC death after several insults and modest protection of ganglion cell layer cells was seen in *Ddit3* null mice after intraocular injection of NMDA^23^. Thus, this ER stress pathway could play an important role in excitotoxic RGC death. Therefore, in order to determine the extent of involvement of DDIT3 in mediating RGC death after excitotoxic injury, mice deficient in *Ddit3* were injected with NMDA and RGC loss analyzed 7 days post-injection. Compared to wildtype, *Ddit3* deficient mice had no significant change in surviving TUJ1+ RGCs after NMDA insult (Fig. 8; *p* = 0.8105 comparing WT to *Ddit3*^−/−^ NMDA). This result suggests the ER stress pathway involving DDIT3 does not play a major role in mediating RGC death after NMDA injury.

**Figure 9:**
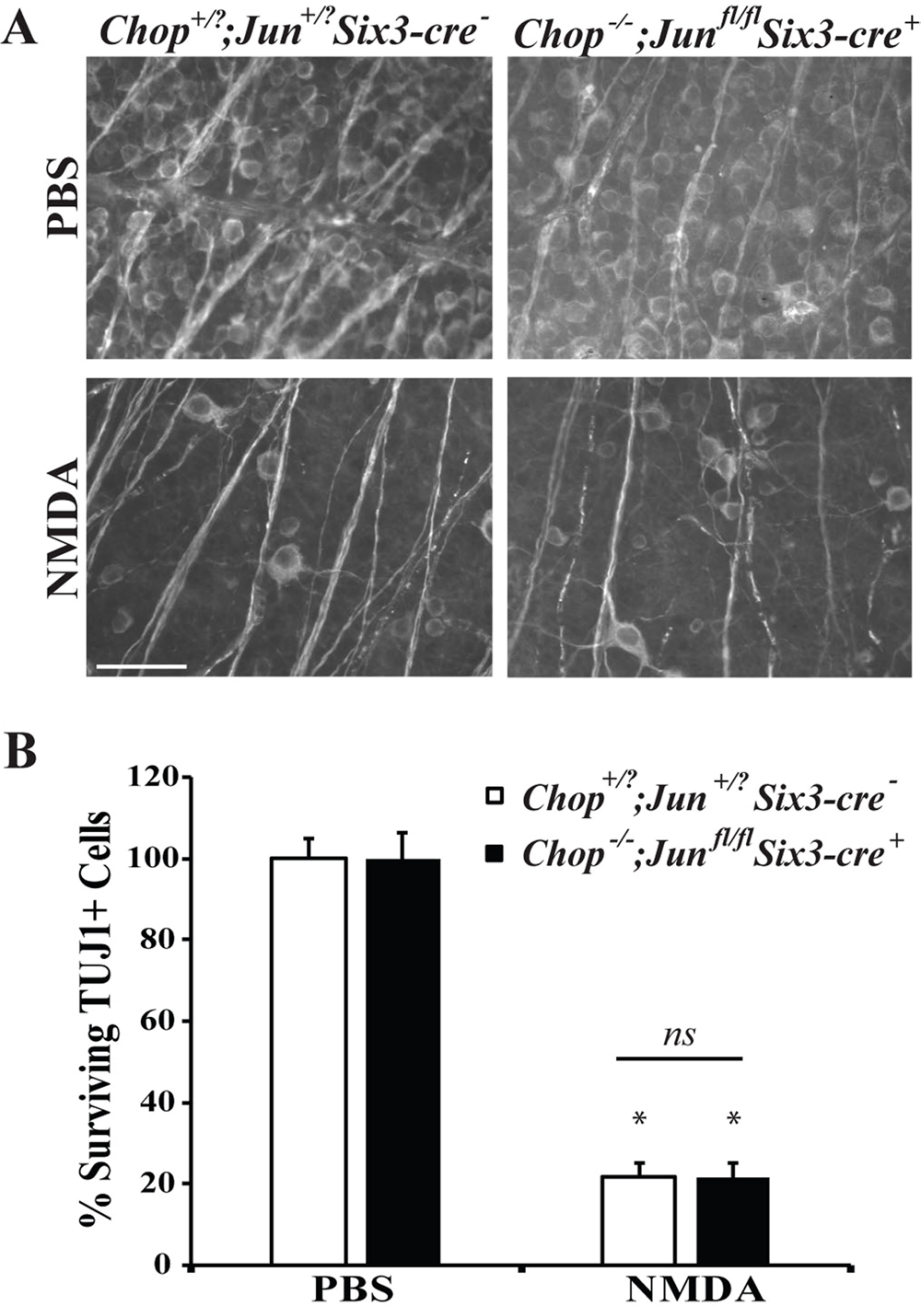
Combined *Jun* and *Ddit3* deficiency does not attenuate loss of RGCs after NMDA insult. (**A**) Representative images showing TUJ1 immunolabeled cells in the GCL of *Jun* and *Ddit3* deficient (*Jun*^*fl/fl*^*Ddit3*^−/−^*; Six3-cre*^+^) mice 7 days after intravitreal NMDA or PBS injection. Significant RGC loss occurred in wildtype (*Jun*^+*/?*^*Ddit3*^+*/?*^*; Six3-cre*^*−*^) as well as *Jun* and *Ddit3* deficient mice after NMDA insult (n = 3 for PBS WT, n = 3 for NMDA WT, n = 4 for PBS *Jun*^+*/?*^*Ddit3*^+*/?*^*; Six3-cre*^*−*^, n = 5 for NMDA *Jun*^+*/?*^*Ddit3*^+*/?*^*; Six3-cre*^*−*^). **(B)** Quantification of TUJ1+ RGCs confirmed that *Jun* and *Ddit3* deficient mice had a similar loss of RGCs compared to wildtype mice 7d after NMDA injection (*p < 0.001 comparing PBS to NMDA; *p* = 0.8582 between WT and *Jun*^*fl/fl*^*Ddit3*^−/−^*; Six3-cre*^+^ NMDA, *ns* between genotypes). Scale bar: 50 μm.

*Jun* or *Ddit3* deficiency alone did not prevent RGC loss after excitotoxic injury, however it has been shown that deficiency in *Jun* and *Ddit3* together provide increased protection to RGC somas after an axonal injury^44^. To test whether these two pathways together mediate RGC loss after NMDA insult, surviving RGCs were counted in mice deficient in both *Jun* and *Ddit3* (*Jun*^*fl/fl*^*Ddit3*^−/−^*; Six3-cre*^+^) after an excitotoxic injury. Mice deficient in both *Jun* and *Ddit3* had a similar amount of RGC loss as wildtype mice 7 days post-NMDA injection (Fig. 9; *p* = 0.8582 between WT and *Jun*^*fl/fl*^*Ddit3*^−/−^*; Six3-cre*^+^ NMDA).

### Kainic acid induced RGC death is not mediated by Jun or Ddit3

**Figure 10:**
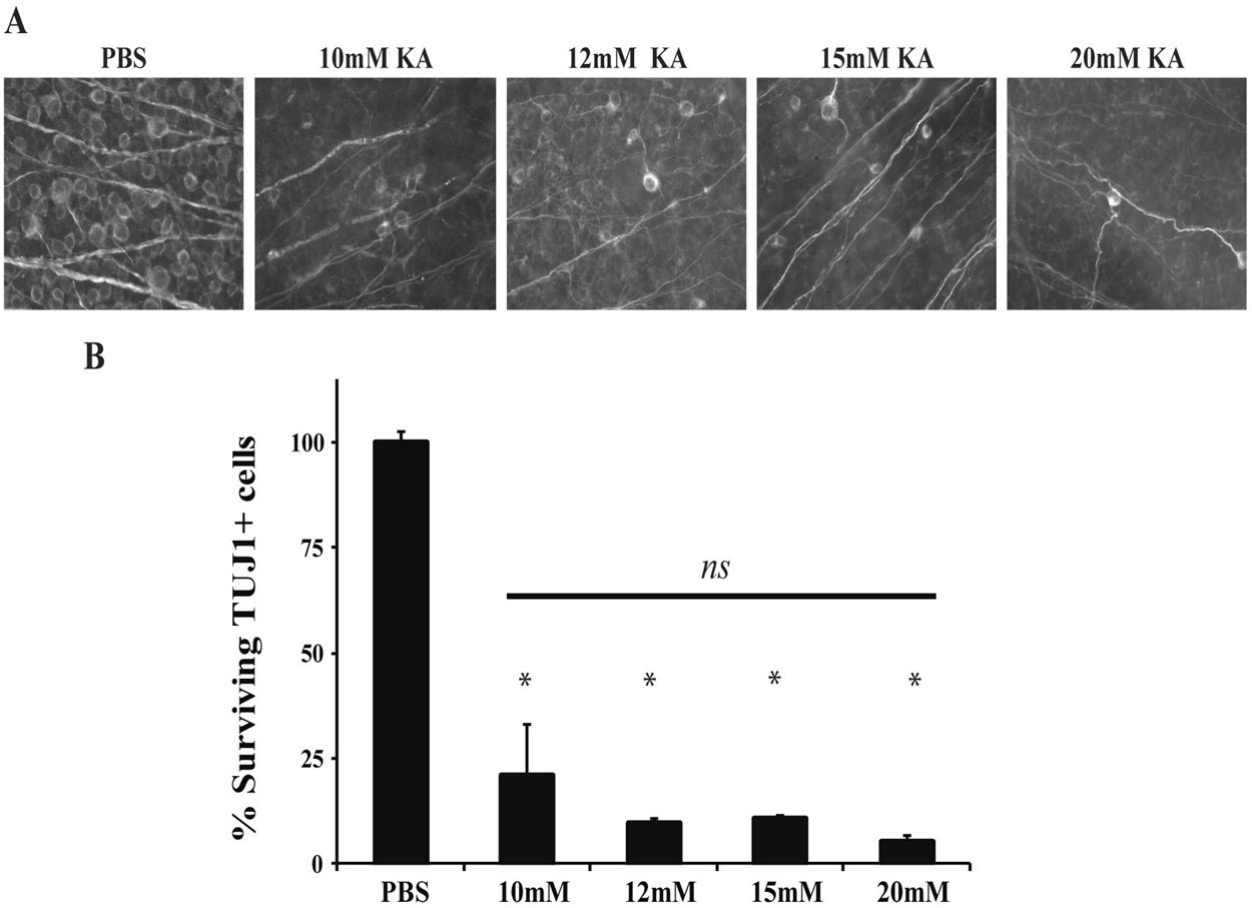
KA induced excitotoxic injury kills RGCs in a dose independent manner. RGC loss was quantified 7 days after intravitreal injection of KA. **(A,B)** The RGC specific marker, TUJ1, was used to label RGCs in flat-mounted retinas 7 days after a 2 μl intravitreal injection of either PBS (vehicle control) or 10 mM, 12 mM, 15, or 20 mM of KA into C57BL/6 J mice (n = 14 for PBS control, n = 6 for 10 mM KA, n = 4 for 12 mM KA, n = 5 for 15 mM KA, n = 4 for 20 mM KA). Quantification of TUJ1 + RGCs showed that KA insult caused a dose independent loss of RGCs. All KA concentrations caused a significant decrease in RGC number (*p < 0.001 for all concentrations compared to control). Scale bar: 50 μm.

**Figure 11:**
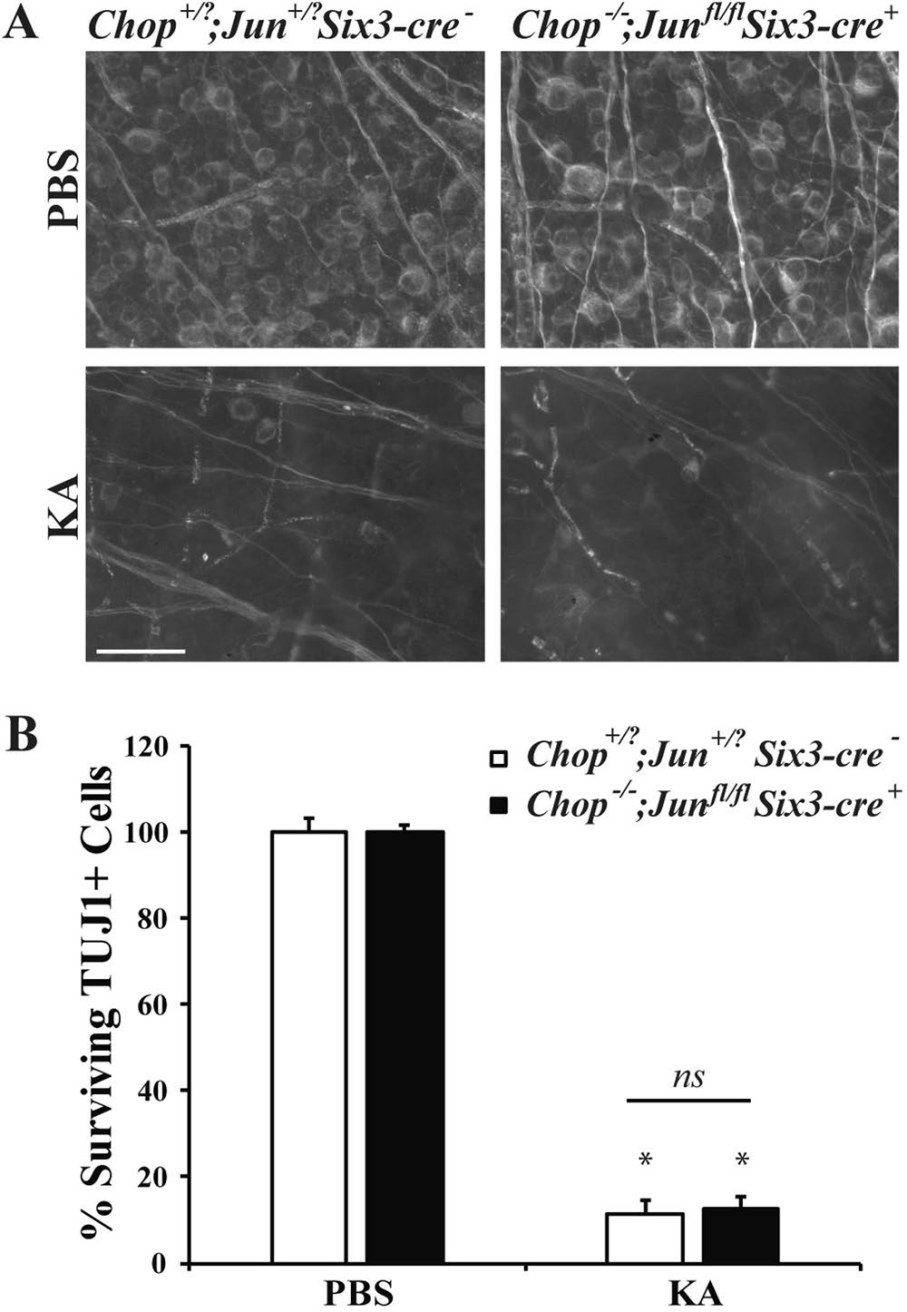
Combined *Jun* and *Ddit3* deficiency does not attenuate loss of RGCs after KA insult. **(A)** Representative images showing TUJ1 immunolabeled cells in the GCL of *Jun* and *Ddit3* deficient (*Jun*^*fl/fl*^*Ddit3*^−/−^; *Six3-cre*^+^) mice 7 days after intravitreal KA or PBS injection. Significant RGC loss occurred in wildtype (*Jun*^+*/?*^*Ddit3*^+*/?*^*; Six3-cre*^*−*^) as well as *Jun* and *Ddit3* deficient mice after KA insult (n = 5 for PBS WT, n = 3 for NMDA WT, n = 4 for PBS *Jun*^+*/?*^*Ddit3*^+*/?*^*; Six3-cre*^−^, *n* = 3 for NMDA *Jun*^+*/?*^*Ddit3*^+*/?*^*; Six3-cre*^*−*^). **(B)** Quantification of TUJ1+ RGCs confirmed that *Jun* and *Ddit3* deficient mice had a similar loss of RGCs compared to wildtype mice 7d after NMDA injection (*p < 0.001 comparing PBS to KA; *p* = 0.9962 between WT and *Jun*^*fl/fl*^*Ddit3*^−/−^*; Six3-cre*^+^ KA, *ns* between genotypes). Scale bar: 50 μm.

There are differences in the severity and specificity of cell death based upon the type of excitotoxin^45^. Another excitotoxin that is used to induce excitotoxic cell death is kainic acid^45–47^. Kainic acid, along with NMDA, is used for investigating neuroprotective strategies to limit RGC loss in glaucoma, retinal ischemia, and diabetic retinopathy^48–50^. *Jun* and *Ddit3* have been shown to be important in KA-mediated death^26,37,51^, thus it is possible that in the retina these molecules are necessary for RGC death induced by KA, but not NMDA. In order to find a concentration that produces consistent and reproducible RGC death, different concentrations of KA were intravitreally injected into wildtype mice (10 mM, 12 mM, 15 mM, or 20 mM; Fig. 10). These KA concentrations are similar to what has been previously used to induce retinal injury^50,52^. All concentrations of KA produced significant RGC loss compared to PBS injection (Fig. 10; *p* < 0.001 for all concentrations compared to control), though the RGC loss achieved was not statistically significant between KA concentrations. Therefore, to assess the involvement of *Jun* and *Ddit3* in KA-induced RGC death, 15 mM KA was intravitreally injected into mice deficient in both *Jun* and *Ddit3* (*Jun*^*fl/fl*^*Ddit3*^−/−^*; Six3-cre*^+^). Surviving RGCs (TUJ1+ cells) were assessed 7 days post injection and there was no statistical difference between KA-injected wildtype and *Jun/Ddit3* deficient mice (Fig. 11; *p* = 0.9962 between WT and *Jun*^*fl/fl*^*Ddit3*^−/−^*; Six3-cre*^+^ KA). This result is similar to what was described above with NMDA-mediated RGC injury. Overall, these data suggest that the JUN/JNK signaling pathway does not play a role in either NMDA or KA-induced excitotoxic RGC death.

## Discussion

In this study, the importance of key components of the extrinsically activated mitochondrial cell death pathway for RGC death after excitotoxic injury were tested. The absence of *Tnf* or *Bid* did not lessen RGC death after excitotoxic insult. The importance of intrinsic cell death signaling pathways was also tested using mouse mutants of various components of the JNK-JUN signaling pathway (*Dlk*, *Jnk2*, *Jnk3* and *Jun*) and ER stress pathway (*Ddit3*). Finally, we tested whether combined deficiency in *Jun* and *Ddit3* provided additive protection to RGCs after excitotoxic insult. Collectively, our data show key extrinsic and intrinsic pathways that have been implicated in RGC death do not appear to be necessary for RGC death after excitotoxic injury.

TNF has been implicated in RGC death after excitotoxic insult^7^. Surprisingly, in our hands *Tnf* deficiency did not provide long-term protection to RGCs after excitotoxic injury nor did it prevent RGC loss at an early time point after insult. Additionally, absence of *Bid* failed to prevent RGC loss after NMDA insult. Together, these data show a key extrinsic pathway that has been implicated in RGC death does not appear to be necessary for mediating RGC death after excitotoxic injury. Lebrun-Julien and colleagues previously showed a significant, but incomplete protection against RGC death in *Tnf* deficient mice six hours after an intravitreal NMDA injection^7^. These experiments are inconsistent with the work presented here showing no protection provided by *Tnf* deficiency at six hours after intravitreal injection of NMDA^7^. It is unclear why the experiments presented here, performed with similar concentrations of NMDA, similar procedures, and both using TNF deficient mice obtained different results compared to Lebrun-Julien and colleagues previous work^7^. However, the longest time point examined by Lebrun-Julien *et al*. was only 6 hours after injury, and at this time point only a partial protection was observed (about half of the cells that had died in the control were protected). It is important to note that Lebrun-Julien and colleagues^7^ found similar protection at this time point using a TNF neutralizing drug, Etanercept. Therefore, it is possible that slight differences in the kinetics of cell loss between the different experiments explain the observed differences. Nevertheless, the experiments described here show that TNF signaling is not required for RGC death after an excitotoxic insult.

Multiple intrinsic pro-death signaling pathways have been implicated in excitotoxic induced RGC death. JNK/JUN signaling has been shown to be involved in excitotoxic neuronal death in multiple systems. Manipulating upstream regulators of JUN have been shown to reduce RGC death after excitotoxic injury, for example *Jnk3* deficiency lessened neuronal loss after a kainic acid-induced excitotoxic injury^37^. Also, an upstream kinase of JNK which is known to activate JNK in injured RGCs^35,53,54^, DLK, has been shown to be involved in excitotoxic insult induced neuronal death^33,35^. In RGCs, multiple lines of evidence point to JNK/JUN signaling having a role in cell death after an excitotoxic insult. JNK and JUN are activated in RGCs after induced excitotoxicity (Fig. 4)^18,22^. Also, inhibiting JNK signaling by an intravitreal injection of a JNK inhibitor decreased RGC loss after NMDA insult^22^. However, despite the potential importance of JNK signaling in excitotoxic injured neurons, experiments herein which critically tested key components of JNK/JUN signaling showed that this pathway may not be a primary driver of excitotoxic RGC death. Specifically, we showed that deficiency in *Jun*, *Jnk2/3*, and *Dlk* did not prevent excitotoxicity-induced RGC death. It is unclear why the results presented here differ with previous studies showing protection from excitotoxic injury after inhibiting the JNK/JUN signaling pathway^20–22^. Two previous studies used pharmaceutical approaches to inhibit JNK signaling^20,22^. For these studies it is possible that there was inhibition of other kinases that contributed to the protection^55^. Munemasa and colleagues specifically tested the importance of JUN activation in NMDA induced RGC death by intravitreally injecting *Jun* anti-sense oligodeoxynucleotides^21^. It is possible that this experiment also resulted in off target effects. Furthermore, all of these experiments counted total ganglion cell layer neurons and not just RGCs. The ganglion cell layer in the mouse consists of approximately equal numbers of RGCs and amacrine cells^56^ and both cell types die after excitotoxic insult^28^. Thus, it is possible that there is protection of amacrine cells that explains the differences between the studies. It is also possible that the genetic deletion of JNK signaling molecules during development alters the response of RGCs to excitotoxic insult.

The lack of protection observed in this study by components of the JNK/JUN signaling pathway is an intriguing result given the known importance of JNK/JUN signaling in RGCs after other insults, particularly after axonal injury^38,57–59^. Thus, these results suggest that in RGCs different molecular pathways are activated depending on the insult. Furthermore, given the potential importance of excitotoxicity in diabetes and ischemic optic neuropathy and axonal injury in glaucoma it suggests that RGCs die by different mechanisms in common human diseases. It is important to note that the experiments described here have not ruled out the potential importance of a JNK1 contribution, particularly a JUN-independent JNK1 function, in mediating RGC death after an excitotoxic insult. It will be important in the future to test the role of JNK1 and even the simultaneous inhibition of all three JNKs to fully define the importance of JNK signaling in excitotoxicity induced RGC death.

After an excitotoxic insult, ER stress components are upregulated and active in RGCs, including *Ddit3*^25^. Furthermore, *Ddit3* deficiency has been shown to protect about 10 cells/mm in the ganglion cell layer more than in C57BL/6 J after an excitotoxic injury^23^. *Ddit3* deficiency did not provide protection to RGCs 7 days after insult, suggesting it is not required for RGC death after and excitotoxic insult. Both JNK/JUN and ER stress signaling contribute to RGC death after axonal injury^27,38^. Recently, our group showed that these pathways appeared to be independently regulated in RGCs after axonal injury^44^. In fact, absence of *Jun* and *Ddit3* provided robust, long-term protection to RGCs after an axonal insult^44^. Thus, it is possible that both of these pathways could contribute to RGC death after an excitotoxic insult and that each is sufficient to kill RGCs. To critically test this hypothesis, RGCs were counted from mice deficient in both *Jun* and *Ddit3* after an excitotoxic insult. Deficiency of both *Jun* and *Ddit3* failed to provide protection to RGCs after intravitreal NMDA injection 7 days after insult. Furthermore, RGC loss after intravitreal injection of kainic acid was not lessened by *Jun/Ddit3* deficiency. Together these data show that JUN and DDIT3 dependent pathways are not required to prevent RGC death after an excitotoxic injury. This result was surprising given that both *Jun* and *Ddit3* are known RGC pro-death molecules and are expressed in RGCs after an excitotoxic injury.

Studies have reported that manipulating various pathways provide protection of RGCs^7,21–23,60^. However, most of these studies have shown only a minor or short-term protection, especially those targeting parts of the pathway downstream of glutamate receptor activation. Furthermore, to date, no one method of intervention has shown complete protection from excitotoxic RGC death. These studies coupled with the results described here indicate that multiple, independent pro-death pathways are active after an excitotoxic insult. This potentially makes preventing excitotoxic RGC death very difficult and designing therapeutic targets challenging in chronic diseases where excitotoxicity has been implicated. Simply inhibiting NMDA and KA channels for the duration of the chronic diseases are likely be detrimental to the system or become ineffective. Additionally, NMDA receptor antagonists failed to show efficacy in multiple clinical trials for stroke and TBI, relegating development of potential therapeutics to downstream pathogenic signaling pathways^61^. Thus, multiple pro-death pathways may need to be targeted to prevent vision loss in diseases were excitotoxicity contributes to RGC death. In the future, it will be important to define all of the pro-death pathways active in RGCs after excitotoxic insult in order to identify  common upstream regulators of these pathways. It is important to note that this study used a typical procedure of giving a single intravitreal injection of a fairly large amount of NMDA or KA. It is unlikely that this type of injury is what occurs during disease. To understand the signaling pathways that control excitotoxic RGC death in human diseases it will be important to model the precise nature of the excitotoxic insult in terms of both concentration and length of insult. Therefore, to properly model disease relevant exposures to excitotoxins, experiments should be performed where excitotoxins are chronically delivered to the retina at low or varying levels.

## Materials and Methods

### Mice

Mice carrying null alleles of *Tnf (Tnf*^*tm1Gkl*^), *Bid* (*Bid*^*tm1Sjk*^), *Jnk2* (*Mapk9*^*tm1Flv*^) and *Jnk3* (*Mapk10*^*tm1Flv*^), or *Ddit3* (*Ddit3*^*tm2.1Dron*^) were obtained from the Jackson Laboratory. Additionally, floxed alleles of *Dlk* (*Dlk*^*fl*^)^62^; and *Jun* (*Jun*^*fl*^)^40^; were used. Floxed alleles were recombined using a retinal expressed cre, Tg(Six3-cre)69Frty mice (referred to as Six3-cre)^41^ Note a “?” for an allele denotes that for controls some animals carried a WT allele while others carried either a null or floxed allele as appropriate (e.g.+/? means both +/+ and +/− genotypes were used). All colonies were maintained by intercrossing. All alleles were on a C57BL/6 J genetic background except for the *Jnk2* and *Jnk3* cross which was on a mixed C57BL/6 J and DBA/2 J genetic background. Mice were housed in a 12-hour light dark cycle and were fed chow and water ad libitum. All mice used were adults (2–5 months of age). All experiments were conducted in accordance with the Association for Research in Vision and Ophthalmology’s statement on the use of animals in ophthalmic research and were approved by the University of Rochester’s University Committee on Animal Resources.

### Intraocular Injection

Mice were anaesthetized with an intraperitoneal injection of 0.05 ml/10 g solution containing ketamine (20 mg/mL) and xylazine (2 mg/mL). A small incision was made with a 30-gauge needle behind the limbus at the superiotemporal quadrant through the conjunctiva at a 45° angle. 2 mM, 20 mM, and 80 mM concentrations of N-Methyl-D-aspartate (NMDA; Sigma) and 10 mM, 12 mM, 15 mM, and 20 mM KA were made in sterile 0.1 M PBS. 2 μl of either NMDA or KA concentrations were injected into the vitreous through the sclera just behind the limbus, using a 5 μL Hamilton syringe equipped with a 33-gauge removable needle. The contralateral eye was injected with 2 μL of vehicle (0.1 M PBS) to serve as a control. To reduce an increase in intraocular pressure, the intravitreal injections were conducted over 2 minutes. The condition of the injected eyes was monitored to ensure no problems arose due to the injection (e.g. cataract).

### Immunohistochemistry and cell counts

RGC survival was assessed using the RGC marker TUJ1, whose expression is sustained following injury^63–65^. Eyes were processed as previously described^38,64^. Following fixation in 4% paraformaldehyde (PFA), the anterior segment of each eye was removed and the posterior eyecup was processed for whole mount immunostaining or cryosectioning. For whole mount immunostaining, retinas were blocked in 0.4% Triton X-100 in PBS containing 10% horse serum for 3–4 hours. Retinas were then incubated in primary antibody against mouse anti-βIII tubulin (mouse anti-TUJ1, BioLegend, 1:1000) diluted in 0.3% Triton X-100 in PBS for 3 days at 4 °C. Following washes in PBS, the retinas were incubated with Alexa Fluor conjugated secondary antibodies (Invitrogen, 1:1000) diluted in PBS for 1 day at 4 °C and then mounted ganglion cell layer up onto slides. RGC density varies greatly with respect to retinal location. For each retina, images were obtained from eight 40× fields around the peripheral retina (two from each quadrant), each field approximately 220 μm from the peripheral edge of the retina (one half of a 40× field in from the peripheral margin). The numbers of neurons immunolabeled with TUJ1 in each image were quantified using the cell-counter tool in ImageJ (NIH). Counts from each field were summed. For immunohistochemistry on retinal sections, cryosections were blocked by incubating in 10% horse serum in 0.1% Triton X-100 in PBS (PBST) for 2–3 h at room temperature. Sections were incubated with primary antibodies (mouse anti-TUJ1, BioLegend, 1:1000; rabbit anti-pJUN, Cell Signaling, 1:250) diluted in PBST overnight at 4 °C. The following day the sections were washed and incubated with Alexa Fluor-conjugated secondary antibodies (Invitrogen, 1:1000) diluted in PBST for a minimum of 2 hours. Sections were then counterstained with 4′,6-diamidino-2-phenylindole (DAPI; Fisher Scientific). For terminal deoxynucleotidyl transferase-mediated dUTP end labeling (TUNEL), mice were euthanized and cryosections were prepared as described above. TUNEL assays were performed according to the manufacturer’s instructions (ApoTag, EMD Millipore).

### Statistical analysis

During quantification of results, the experimenter was masked to genotype and/or experimental group. Experiments with two groups were analyzed for differences using a one-way ANOVA and three or more groups, either multiple time points or genotypes, underwent statistical analyses using a two-way ANOVA with significance determined at *P* values < 0.05, followed by the Tukey’s or Sidak’s multiple comparisons test for group comparisons.
